# Supplementary figures and images for: LINC00958 promotes proliferation, migration, invasion, and epithelial-mesenchymal transition of oesophageal squamous cell carcinoma cells
Source: PLoS One. 2021 May 18;16(5):e0251797. doi: 10.1371/journal.pone.0251797 (PMC8130937; doi:10.1371/journal.pone.0251797)

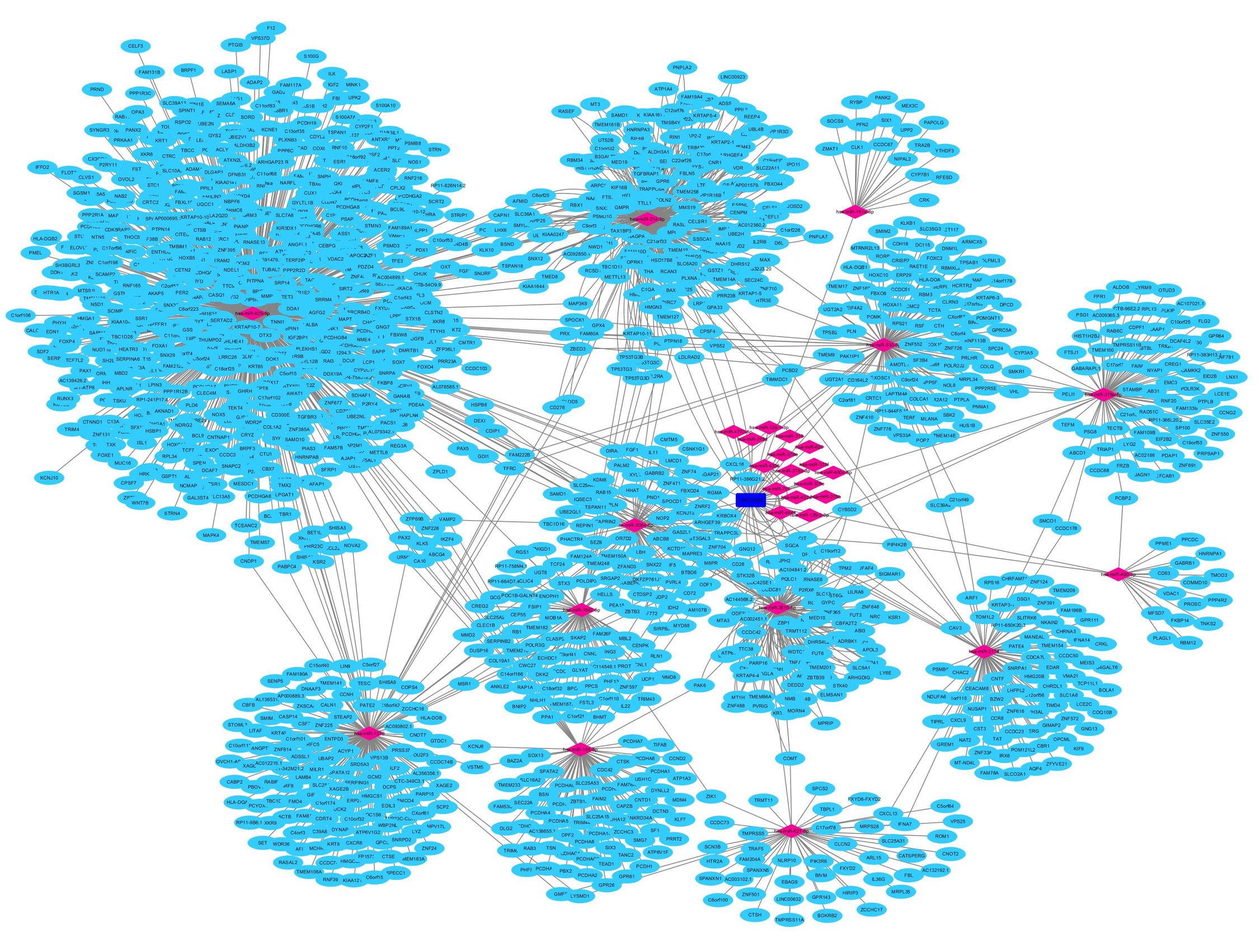

Supplement: S1 Fig — (TIF) [file pone.0251797.s001.tif]

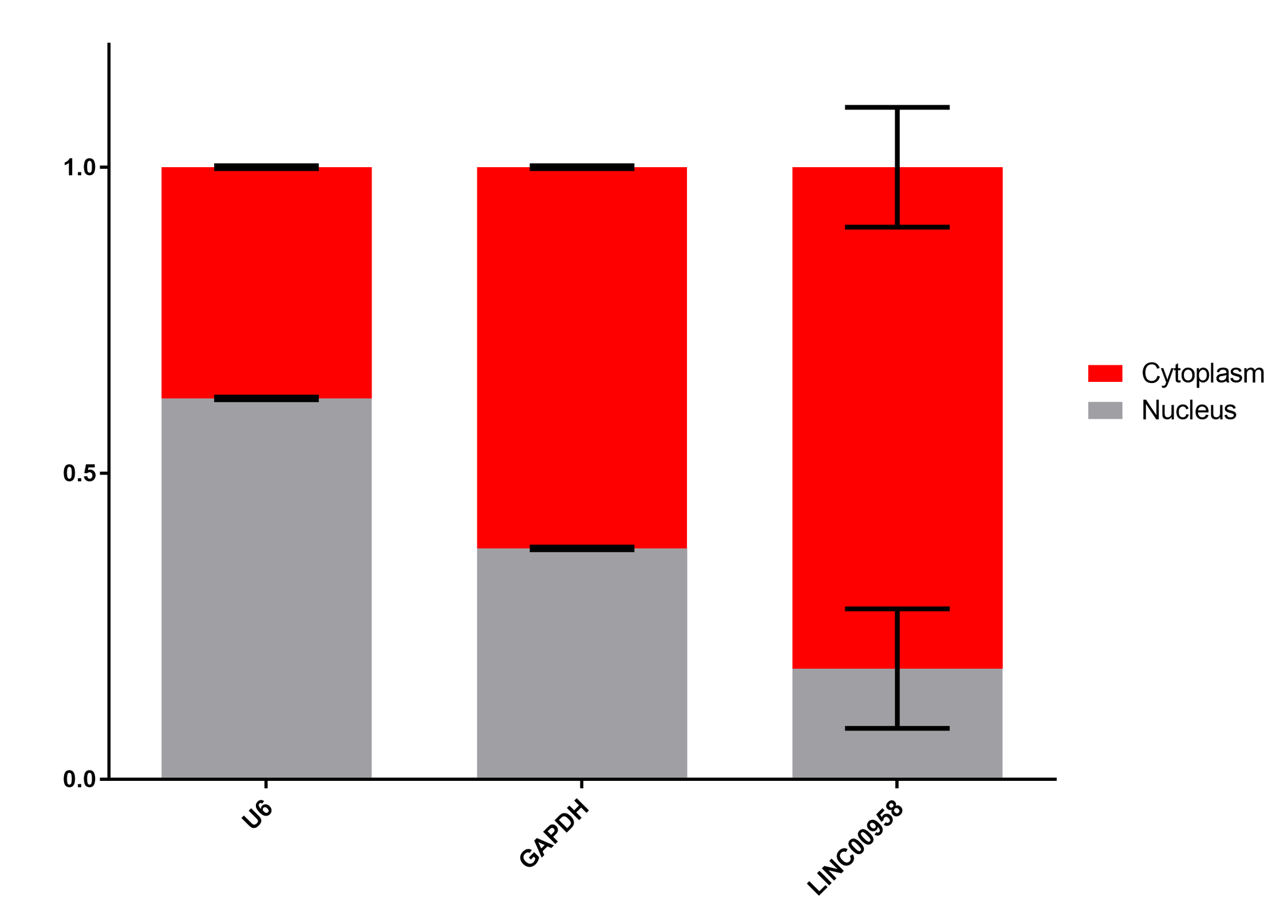

Supplement: S2 Fig — (TIF) [file pone.0251797.s002.tif]

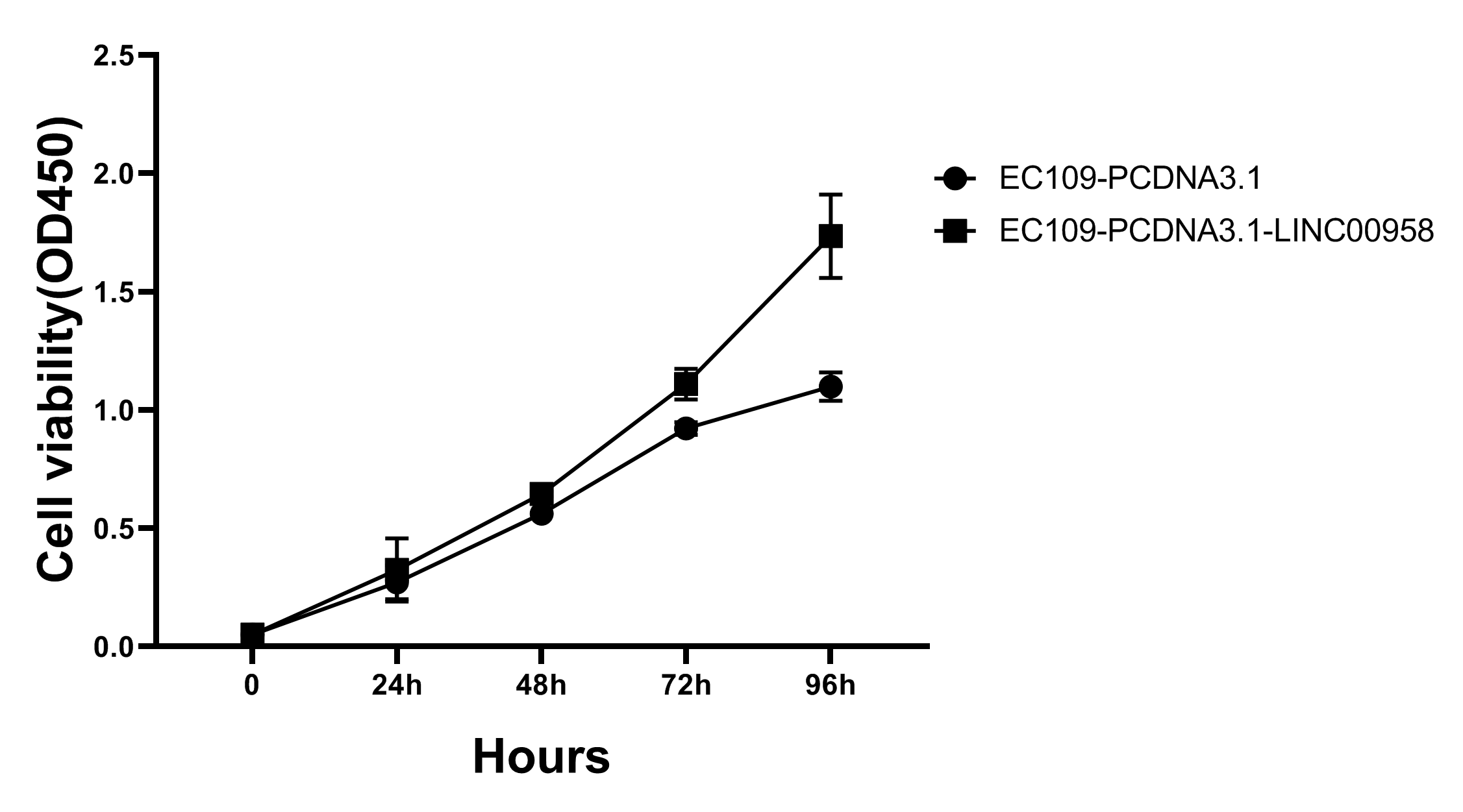

Supplement: S3 Fig — (TIF) [file pone.0251797.s003.tif]
