# Supplementary material for: LINC00958 promotes proliferation, migration, invasion, and epithelial-mesenchymal transition of oesophageal squamous cell carcinoma cells
Source: PLoS One. 2021 May 18;16(5):e0251797. doi: 10.1371/journal.pone.0251797 (PMC8130937; doi:10.1371/journal.pone.0251797)
Supplement: S1 Raw images — (PDF) [file pone.0251797.s006.pdf]

Original images for blots:

EC109 cells:

1.

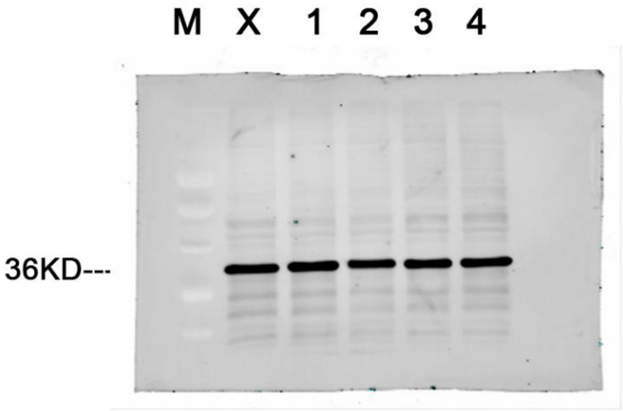

Protein: GAPDH
M: marker(Thermo Scientific™ 26616)
1: siRNA-N
2: siRNA-LINC00958
3: pcDNA3.1
4: pcDNA3.1-LINC00958

Method used to capture the image:

Odyssey infrared imaging system (LI-COR Biosciences, Lincoln, NE, USA)

Figure panel: Fig 3E and Fig 3G GAPDH(upper).

2.

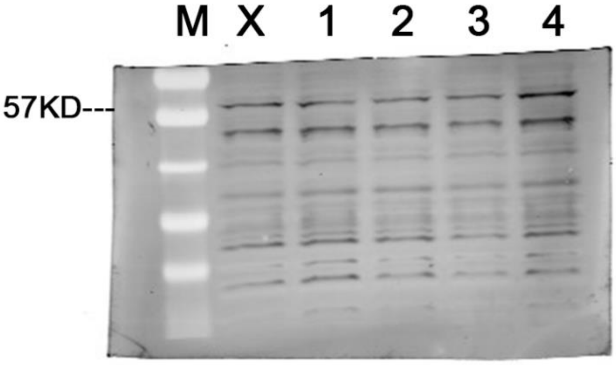

Protein: Vimentin
M: marker(Thermo Scientific™ 26616)
1: siRNA-N
2: siRNA-LINC00958
3: pcDNA3.1
4: pcDNA3.1-LINC00958

Method used to capture the image:

Odyssey infrared imaging system (LI-COR Biosciences, Lincoln, NE, USA)

Figure panel: Fig 3E and Fig 3G Vimentin

3.

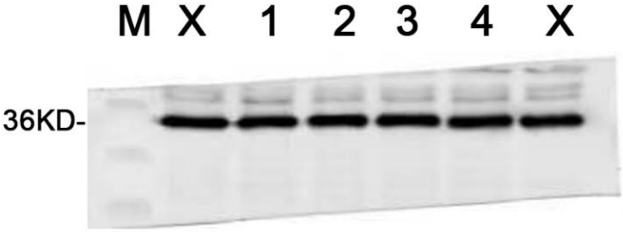

Protein: GAPDH
M: marker(Thermo Scientific™ 26616)
1: pcDNA3.1
2: pcDNA3.1-LINC00958
3: siRNA-N
4: siRNA-LINC00958

Method used to capture the image:

Odyssey infrared imaging system (LI-COR Biosciences, Lincoln, NE, USA)

Figure panel: Fig 3E and Fig 3G GAPDH (lower).

Original images for blots:

EC109 cells:

4.

M X 1 2 3 4 X

135KD--

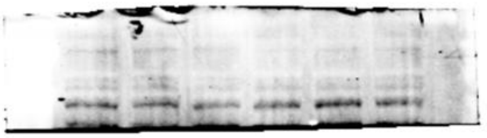

Protein: E-Cadherin
M: marker (Thermo Scientific™ 26616)
1: pcDNA3.1
2: pcDNA3.1-LINC00958
3: siRNA-N
4: siRNA-LINC00958

Method used to capture the image:

Odyssey infrared imaging system (LI-COR Biosciences, Lincoln, NE, USA)

Figure panel: Fig 3E and 3G E-Cadherin

5.

M X 1 2 3 4 X

59KD--

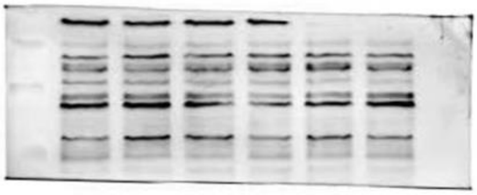

Protein: Occludin
M: marker (Thermo Scientific™ 26616)
1: pcDNA3.1
2: pcDNA3.1-LINC00958
3: siRNA-N
4: siRNA-LINC00958

Method used to capture the image:

Odyssey infrared imaging system (LI-COR Biosciences, Lincoln, NE, USA)

Figure panel: Fig 3E and 3G Occludin

6.

M X 1 2 3 4 X

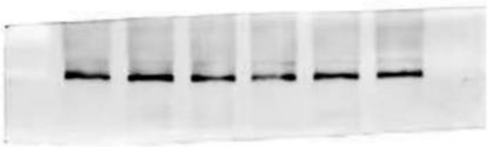

Protein: ZO-1
M: marker (EpiZyme WJ102L)
1: pcDNA3.1
2: pcDNA3.1-LINC00958
3: siRNA-N
4: siRNA-LINC00958

Method used to capture the image:

Odyssey infrared imaging system (LI-COR Biosciences, Lincoln, NE, USA)

Figure panel: Fig 3E and 3G ZO-1

Original images for blots:

EC109 cells:

7.

M X 1 2 3 4

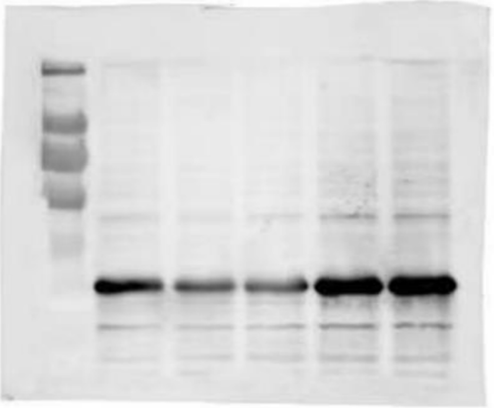

36KD--

Protein: GAPDH

M: marker (Thermo Scientific™ 26616)

1: pcDNA3.1

2: pcDNA3.1-LINC00958

3: siRNA-N

4: siRNA-LINC00958

Method used to capture the image:

Odyssey infrared imaging system (LI-COR Biosciences, Lincoln, NE, USA)

Figure panel: Fig 4D and 4E GAPDH

8.

M X 1 2 3 4

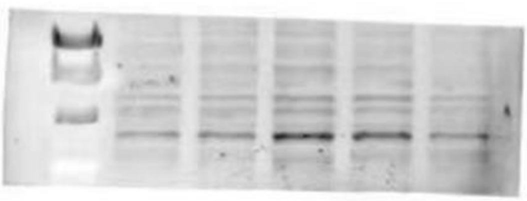

48KD--

Protein: SPOCK1

M: marker (Thermo Scientific™ 26616)

1: pcDNA3.1

2: pcDNA3.1-LINC00958

3: siRNA-N

4: siRNA-LINC00958

Method used to capture the image:

Odyssey infrared imaging system (LI-COR Biosciences, Lincoln, NE, USA)

Figure panel: Fig 4D and 4F SPOCK1

Original images for blots:

EC109 cells:

9.

M X XX X X 1 2 3

36KD--

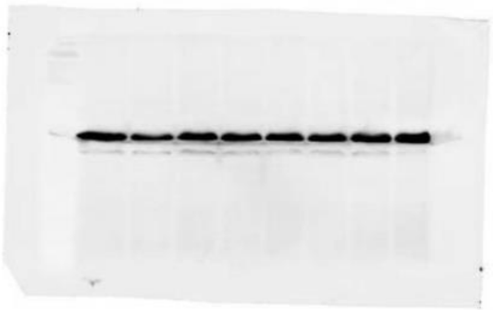

Protein: GAPDH

M: marker (Thermo Scientific™ 26616)

1: NC

2: miR-510-5p 50 pmol

3: miR-510-5p 100 pmol

Method used to capture the image:

Odyssey infrared imaging system (LI-COR Biosciences, Lincoln, NE, USA)

Figure panel: Fig 4H GAPDH

10.

M X XX X 1 2 3

48KD-

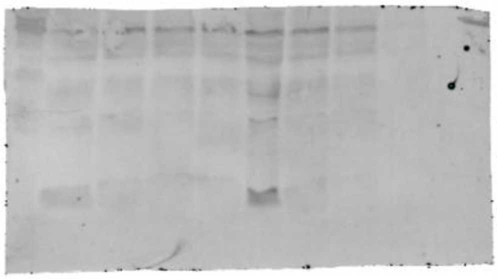

Protein: SPOCK1

M: marker (Thermo Scientific™ 26616)

1: NC

2: miR-510-5p 50 pmol

3: miR-510-5p 100 pmol

Method used to capture the image:

Odyssey infrared imaging system (LI-COR Biosciences, Lincoln, NE, USA)

Figure panel: Fig 4H SPOCK1
