# Supplementary material for: LINC00958 promotes proliferation, migration, invasion, and epithelial-mesenchymal transition of oesophageal squamous cell carcinoma cells
Source: PLoS One. 2021 May 18;16(5):e0251797. doi: 10.1371/journal.pone.0251797 (PMC8130937; doi:10.1371/journal.pone.0251797)
Supplement: Attachment — Submitted filename: Response to Reviewers.docx [file pone.0251797.s008.docx]

Dear Dr. Andl and reviewers,

Re: Resubmission of manuscript reference PONE-D-20-41067R1.

Thank you for inviting us to submit a revised version of the manuscript entitled, " LINC00958 upregulates SPOCK1 expression to promote the development of oesophageal squamous cell carcinoma by sponging miR-510-5p" to PLOS ONE. Thank you for your approval of our previous revision, we also appreciate the time and effort you have dedicated to providing insightful feedback on ways to strengthen our paper. Thus, it is with great pleasure that we resubmit our article for further consideration. We have incorporated changes that reflect the detailed suggestions you have graciously provided. The title has now been changed to “LINC00958 promotes proliferation, migration, invasion, and Epithelial-Mesenchymal Transition of oesophageal squamous cell carcinoma cells”. We also hope that our revision and the responses we provide below satisfactorily address all the issues and concerns you and the reviewers have noted. If there is anything inappropriate in the revision, please point it out and we will revise it seriously.

Thank you for your consideration. We look forward to hearing from you.

Kind Regards,

Biqi Wang, MA.Eng, +86 18811071427

Faculty of environment and life, Beijing University of Technology, Beijing 100124, China

Email: 1585835414@qq.com

To facilitate your review of our revisions, the following is a point-by-point response to the questions and comments delivered in your letter dated April 27, 2021.

**Journal Requirements:**

1. ***[Please review your reference list to ensure that it is complete and correct. If you have cited papers that have been retracted, please include the rationale for doing so in the manuscript text, or remove these references and replace them with relevant current references. Any changes to the reference list should be mentioned in the rebuttal letter that accompanies your revised manuscript. If you need to cite a retracted article, indicate the article’s retracted status in the References list and also include a citation and full reference for the retraction notice.]***

**RESPONSE:** We have reviewed the reference list and no retracted papers were cited in the manuscript. The changes to the reference list are as follows:

1. We’ve rescinded the use of the first quotation, and replace it with the current citation 1. Sung H, Ferlay J, Siegel RL, Laversanne M, Soerjomataram I, Jemal A, et al. Global cancer statistics 2020: GLOBOCAN estimates of incidence and mortality worldwide for 36 cancers in 185 countries. CA Cancer J Clin. 2021.countries. due to the update of data. (page 2, line 41)
2. We’ve removed the previous citation [23] and change it to citation [39] which is cited in the discussion. (page 16, line 340)

**Additional Editor Comments:**

1. ***[However, point 4 of the PLOS ONE publication criteria require that conclusions are supported by the data and this point the authors’ conclusion that LINC00958 is promoting growth by regulating SPOCK1 expression via its sponging of miR-510-5p is still not well supported by the data. More experimentation would be required to strengthen these claims prior to publication: The newly added data demonstrate that LINC00958 is regulating SPOCK1 expression, but the magnitude is small and further evidence is drawn from the literature and published changes in SPOCK1 in regard to ESCC proliferation and EMT. The authors would be required to demonstrate that SPOCK1 knockdown or overexpression rescue the phenotypes observed as result of LINC00958 knockdown or overexpression. I would like to suggest to instead change the title to focus on the role of LINC00958 in directly measured outcomes.]***

**RESPONSE:** Thank you for your suggestions. We agree with you and have changed the title of the article to “LINC00958 promotes proliferation, migration, invasion, and Epithelial-Mesenchymal Transition of oesophageal squamous cell carcinoma cells”. (page 1, lines 4-6)

As for the question of the small magnitude of LIN00958 regulating the expression of SPOCK1, it have intuitively shows that the overexpression of LINC00958 significantly increases the expression of SPOCK1, while the knockdown of LINC00958 significantly reduces the expression of SPOCK1 in Fig. 4D and 4F. We used the ImageJ software again to conduct quantitative analysis on Fig. 4D 4F, and the results as shown in Fig. 4E and 4G were obtained.

2. ***[Focus on suggestions from reviewer 1 to:Include a legend for the graph in panel A.]***

**ROSPONSE:**Thank you for your suggestion.We have added the legend for the graph in panel A. (Fig 1A) (pages 10-11, lines 210-212)

3. ***[Change the writing to not overstate the results and drawn conclusion, e.g., LINC00958 regulates migration and the EMT pathway. However, more work is needed to conclude that the observed decrease in migration following LINC00958 modulation is specifically by the EMT pathway. Remove the statements regarding the role of SPOCK1 in ESCC to reflect that the experiments do not address this question.]***

**RESPONSE:** Thank you for your advice. We have changed the writing in the manuscript and have removed the statement regarding the role of SPOCK1 in ESCC. The specific modifications are as follows:

1. The results indicated there was a high expression of LINC00958 in ESCC, which promoted proliferation, migration, invasion and Epithelial–Mesenchymal Transition (EMT) process of ESCC cells, and this effect may be via regulating miR-510-5p. (page 2, lines 35-37)
2. These results indicated that *LINC00958* could regulate migration, invasion and EMT of ESCC.(page 13, lines 256-257)
3. Therefore, we suggested that *LINC00958* might regulate the expression of SPOCK1 by its sponging of miR-510-5p, and involve proliferation, migration, invasion and EMT process of ESCC. (page 16, lines 340-342)

**Reviewer#1 Suggestions:**

[Major concern]

1. ***[One of the central conclusions of the manuscript – that LINC00958 is contributing to ESCC cell proliferation and migration by suppressing SPOCK1 expression via ‘sponging’ of miR-510-p – is not well supported by the data.]***

**RESPONSE:** Thank you for your suggestion. In our manuscript, it can be concluded that LINC00958 can regulate the proliferation and migration of oesophageal squamous cell cancer cells, and LINC00958 can regulate the expression of SPOCK1 by sponging miR-510-5p, but the conclusion that LINC00958 is contributing to ESCC cell proliferation and migration by suppressing SPOCK1 expression via ‘sponging’ of miR-510-p is indeed not sufficiently supported by the data. After our discussion, the title of the manuscript has been changed to " LINC00958 promotes proliferation, migration, invasion, and Epithelial-Mesenchymal Transition of oesophageal squamous cell carcinoma cells".

2. [***While the authors have now added data that demonstrate that LINC00958 is regulating SPOCK1 expression, these data are not particularly convincing given the small (~25%) magnitude of the changes. While the authors argue that changes in SPOCK1 have been well-studied in ESCC and are associated with proliferation and EMT, this evidence likely does not extend to the small changes in SPOCK1 expression observed during LINC00958 over or underexpression. The authors need to address this discrepancy, ideally by determining if SPOCK1 knockdown or overexpression rescue the phenotypes observed by LINC00958 knockdown or overexpression.]***

**RESPONSE:** Thank you for your advice. The western blot results have intuitively shows that the expression of SPOCK1 significantly increased when LINC00958 was overexpressed (Fig 4D), and significantly decreased when LINC00958 was downregulated (Fig 4F). We used the ImageJ software again to conduct quantitative analysis on Fig. 4D and 4F, and get a significant magnitude of change as shown in Fig. 4E (~125%) and 4G (~36%) were obtained. At the same time, we have changed the statement of the conclusion to: “These results indicated that LINC00958 could regulate migration, invasion and EMT of ESCC.” (page 13, lines 256-257) “The results indicated that LINC00958 could regulate the expression of SPOCK1 by competing with miR-510-5p in ESCC.” (page 14, lines 282-283) “Therefore, we suggested that LINC00958 might regulate the expression of SPOCK1 by its sponging of miR-510-5p, and involve proliferation, migration, invasion and EMT process of ESCC.” (page 16,lines 340-342)

**[**Minor concerns:]

1. ***[Panel A – Please include a legend for this graph.]***

**RESPONSE:** Thank you for your suggestion.We have added the legend for the graph in panel A. (Fig 1A) (pages 10-11, lines 210-212)

2. ***[Some conclusions need to be rewritten to be supported by the data:“These results all indicated that LINC00958 could regulate the migration and invasion by the EMT pathway” (line 257-259). These results indicate that LINC00958 regulates migration and the EMT pathway. However, more work is needed to conclude that the observed decrease in migration following LINC00958 modulation is specifically by the EMT pathway. Please discuss.]***

***RESPONSE:*** Thank you for your suggestion. We have rewritten the conclusion to “These results indicated that LINC00958 could regulate migration, invasion and EMT of ESCC.”(page 13, lines 256-257) “We also detected an upregulated expression of LINC00958 in ESCC cells, and verified that LINC00958 promoted proliferation, migration, invasion and EMT process of ESCC.” (page 15,lines 318-320)

3. ***[“Thus, it was clear that SPOCK1 had a cancer-promoting function in ESCC” (285-286).These experiments do not address the role of SPOCK1 in ESCC. Please remove this conclusion.]***

**RESPONSE:** Thank you for your suggestion. We have removed the conclusion about SPOCK1 had a cancer promoting funtion in ESCC, and have rewritten the conclusion to “Therefore, we suggested that LINC00958 might regulate the expression of SPOCK1 by its sponging of miR-510-5p, and involve proliferation, migration, invasion and EMT process of ESCC.” (page 16, lines 340-342)
